# Relationships between physical fitness and early standing-start kinematic characteristics in adolescent male track cyclists

**DOI:** 10.3389/fphys.2026.1948251

**Published:** 2026-10-08

**Authors:** Wei Li, Qian Zhang, Qiang Gao, Delong Dong, Yuwei Guo

**Affiliations:** 1 School of Physical Education, Ludong University, Yantai, China; 2 Department of Traffic Engineering, Shandong Transport Vocational College, Weifang, China; 3 Yantai Sports Park Management and Service Center, Yantai, China

**Keywords:** biomechanics, continuous kinematics, kinematic characteristics, physical fitness, sprint track cycling, standing start

## Abstract

The early standing-start phase is important for sprint track cycling, yet continuous standing-start kinematics and their relationships with physical fitness remain unclear in adolescent athletes. This study characterized standing-start kinematics, assessed their reliability, and examined their associations with physical fitness in 11 adolescent male track cyclists. Participants completed standing long jump, back squat 1RM, and 30-s cycling sprint tests, followed by three standing-start trials. Continuous trunk and lower-limb kinematics from movement initiation through the first 180° of crank rotation were obtained using markerless motion analysis. The three-trial mean showed high reliability across the six kinematic variables (ICC(A,3) = 0.810–0.965). Relative mean power showed the strongest association with knee range of motion (ρ = 0.700, raw p = 0.0165), but none of the 24 associations remained significant after BH-FDR correction. These findings support repeated-trial assessment for reliable characterization of standing-start kinematics and provide preliminary evidence for further investigation of fitness–kinematic relationships in adolescent track cyclists.

## 1 Introduction

Sprint track cycling is a high-intensity discipline characterized by the rapid production of high levels of force and power during acceleration (Dorel et al., 2005; Douglas et al., 2021). Within sprint events, the standing start represents a critical phase in which cyclists must overcome initial inertia and rapidly develop velocity, with early acceleration closely related to subsequent opening-lap performance (Kordi and van Rijswijk, 2024). During the standing start, rapid force and power production is accompanied by coordinated motion of the trunk and lower limbs during initial acceleration (Zhang et al., 2015; Padulo et al., 2016). Standing-start performance therefore reflects not only the capacity to generate high mechanical output but also the organization of movement across multiple body segments. Understanding these biomechanical characteristics is important for characterizing how sprint cyclists execute the early acceleration phase.

The multi-segment nature of the standing start places particular importance on how joint actions are organized throughout the movement. In cycling, coordination is shaped by task-specific constraints, with changes in posture and cadence altering joint mechanics and neuromuscular recruitment across the lower limb (Li, 2004). This organization cannot be fully characterized by considering individual joints in isolation. For example, differences associated with cycling expertise have been observed in the consistency of inter-joint coordination even when mean joint kinematics remain relatively similar (Chapman et al., 2009), while cadence can modify the phase relationships between adjacent lower-limb joints (Sides and Wilson, 2012). Standing-start biomechanics has similarly been characterized through joint motion and other biomechanical variables at specific crank positions or movement events, providing important information on the mechanical organization of start execution (Zhang et al., 2015). However, such discrete measures describe selected features of the movement and provide limited information on how segmental motion develops across the entire movement interval.

This limitation becomes particularly relevant when the temporal evolution of movement is of interest. By retaining kinematic changes across the movement cycle, continuous analysis can complement information obtained from discrete measures at selected events. In cycling, whole-curve analysis has identified kinematic differences that are confined to specific phases of the crank cycle, demonstrating that relevant changes may not be fully represented by selected discrete variables (Galindo-Martínez et al., 2021). Temporal relationships between lower-limb joints also vary with cycling conditions, further indicating that the organization of movement changes across the cycle rather than remaining constant (Sides and Wilson, 2012). Despite these advances in cycling biomechanics, less is known about how trunk and lower-limb kinematics evolve continuously during the early standing-start phase, particularly in adolescent track cyclists. Characterizing these temporal profiles may therefore provide complementary information on the organization of standing-start movement beyond that available from discrete kinematic measures alone. Establishing the consistency of derived kinematic measures across repeated trials is also necessary to distinguish reproducible movement characteristics from trial-to-trial variation.

Beyond the characterization of movement itself, an additional question is whether standing-start kinematic characteristics are related to the physical capacities required for sprint cycling. Muscular power is a major determinant of sprint-cycling performance, with greater power relative to the bicycle–rider system mass being particularly relevant to acceleration from a standing start (Douglas et al., 2021). Evidence from elite track sprinters further demonstrates the importance of maximal power and power–velocity characteristics to sprint performance (Dorel et al., 2005). More specifically, recent evidence in elite sprint track cyclists has shown strong associations between jump performance, short-duration cycling power, and standing-start acceleration performance (Michalik et al., 2026). Strength and rapid force-production capacities may contribute to the mechanical demands of sprint cycling, although their expression on the bicycle depends on the specific requirements of the cycling task (Connolly et al., 2023). This distinction is supported by training evidence showing that improvements in strength can be accompanied by changes in cycling power and intermuscular coordination rather than a uniform change in a particular movement variable (Burnie et al., 2022). Differences in strength capacity have also been associated with distinct patterns of muscle activation across cycling conditions, suggesting that physical capacity may be expressed through neuromuscular organization as well as mechanical output (Bieuzen et al., 2007). Thus, although strength and power are clearly relevant to sprint performance, it remains unclear whether individual differences in these capacities correspond to specific kinematic characteristics during the early standing-start phase.

Accordingly, the present study aimed to characterize continuous sagittal-plane kinematics during the early standing-start phase in adolescent male track cyclists, evaluate the trial-to-trial reliability of selected kinematic measures, and examine their associations with physical fitness. Continuous trunk and lower-limb kinematics were quantified alongside measures of explosive strength, maximal strength, and cycling-specific power. Given the limited evidence directly linking physical capacities to standing-start kinematics, the association analyses were considered exploratory, and no directional hypotheses were specified.

## 2 Materials and methods

### 2.1 Participants

Seventeen male adolescent track cyclists aged 15–18 years were initially recruited from a provincial track cycling team. All athletes had completed at least three years of systematic sprint cycling training and were actively engaged in regular training at the time of testing. The athletes primarily competed in the team sprint. Six athletes were excluded from the kinematic analysis because occlusion in their standing-start recordings prevented sufficiently complete sagittal-plane visualization for consistent landmark extraction. The final analytical sample therefore comprised 11 athletes, all of whom used a right-leg standing-start technique. Among the 11 athletes included in the final analysis, three held Chinese National First-Class Athlete certification, whereas the remaining eight had not attained this classification.

Athletes were eligible if they were actively training, had no musculoskeletal injury or neurological condition that could interfere with testing within the preceding three months, and were able to complete the physical fitness and standing-start assessments. Written informed consent was obtained from all participants and their legal guardians before testing. The study was approved by the Institutional Review Board of Ludong University (approval no. LDU-IRB2026071002) and conducted in accordance with the Declaration of Helsinki.

### 2.2 Experimental protocol

All assessments were completed on the same day. Physical fitness testing was conducted in the morning following a standardized 15-min warm-up consisting of dynamic stretching, mobility exercises, and submaximal cycling. The test order was fixed for all participants: anthropometric measurements, standing long jump, back squat one-repetition maximum (1RM), and 30-s maximal cycling sprint, with at least 10 min of recovery between tests. The morning physical-fitness session and afternoon standing-start session were separated by approximately 3 h, during which participants rested and performed no additional strenuous exercise. Standing-start testing was subsequently conducted in the afternoon following the same standardized 15-min warm-up protocol. Each participant completed three standing-start trials separated by at least 10 min of recovery. All trials were processed independently for reliability analysis and subsequently averaged within each participant for the association analyses (n = 11).

### 2.3 Physical fitness assessment

Body height and mass were measured using a standard stadiometer and digital scale, respectively. Lower-limb explosive strength was assessed using the standing long jump following the ALPHA fitness testing procedures (España-Romero et al., 2010). Participants performed three maximal jumps from behind the starting line with their feet approximately shoulder-width apart on a non-slip surface. The longest valid jump was recorded to the nearest centimeter and retained for analysis.

Maximal lower-limb strength was assessed using a one-repetition maximum (1RM) back squat test (Brown and Weir, 2001). Following warm-up sets at approximately 50% and 70% of the estimated 1RM, the load was progressively increased until the maximum load that could be lifted once with acceptable technique was determined. All attempts were supervised by the same investigator, and repetitions that did not meet the required technique were not accepted. Rest intervals of 1–5 min were provided between attempts. Relative squat strength was calculated as 1RM normalized to body mass.

Maximal cycling power was assessed using a Wattbike Pro cycle ergometer (Wattbike Ltd., Nottingham, UK), which has demonstrated acceptable validity and reliability for cycling power assessment (Hopker et al., 2010). The ergometer was zeroed before testing, and the same fixed resistance setting was used for all participants (air resistance, 10; magnetic resistance, 7). Each participant completed three 30-s maximal sprint trials from a stationary start, with at least 10 min of recovery between trials. No verbal encouragement was provided during the tests, and no separate familiarization session was conducted. Peak and mean power were obtained directly from the Wattbike system. Peak power represented the highest power recorded over a single complete crank revolution, whereas mean power represented the average power over the complete 30-s trial. Peak- and mean-power values were averaged separately across the three trials and subsequently normalized to body mass to obtain relative peak power and relative mean power (W·kg^-^¹).

### 2.4 Standing start testing

Standing-start testing was conducted at the outdoor Shandong Cycling Association Velodrome (Rizhao, China). All participants used the same Pardus T10 track bicycle (Pardus, China), with a frame size of L, a gear ratio of 51:14, and a crank length of 170 mm. Participants adopted their habitual riding positions, and the initial crank position was individually selected according to each athlete’s habitual standing-start preference. Bicycle configuration remained unchanged across the three trials. Each trial was initiated from a starting gate using an electronic start signal. Standing-start performance was recorded using a Sony FX3 camera (Sony Corporation, Tokyo, Japan) at a resolution of 1920 × 1080 pixels and 120 frames·s^-^¹, with a shutter speed of 1/1000 s. The camera was positioned on the left side of the participants, approximately 10 m from the cycling path, with the optical axis oriented perpendicular to the sagittal plane. Each participant completed three valid trials separated by at least 10 min of recovery. A trial was considered valid when the sagittal-plane view remained clear and unobstructed throughout the analyzed movement interval. All three valid trials were retained and processed independently for the trial-to-trial reliability analysis. The camera setup and recording area are illustrated in **Figure 1**.

**Figure 1:**
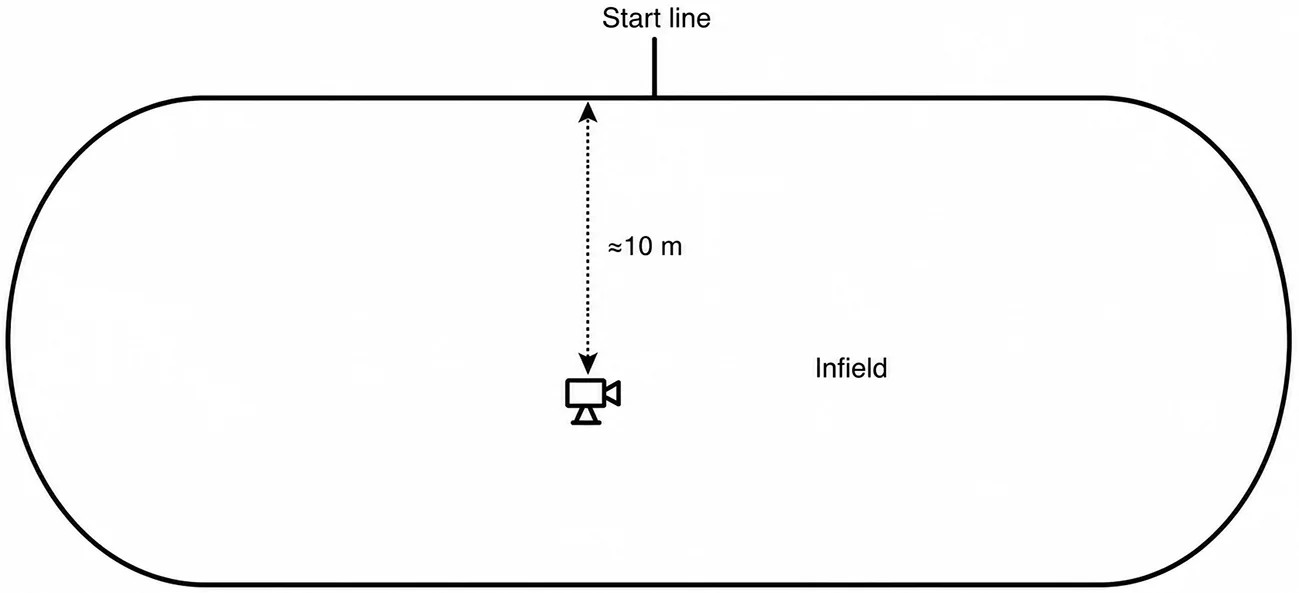
Schematic representation of the camera setup and recording area.

### 2.5 Markerless kinematic analysis

Video data were processed using a top-down markerless pose-estimation pipeline to obtain two-dimensional kinematic data. Athlete detection was performed using YOLOX (Ge et al., 2021), followed by body pose estimation using the RTMPose-m model (Jiang et al., 2023), with model inference implemented using the ONNXRuntime backend. The left shoulder, hip, knee, and ankle landmarks were extracted from each frame. Landmark coordinates with confidence scores <0.30 were treated as missing, and gaps of up to three consecutive frames were linearly interpolated. Trials with unresolved missing coordinates were visually reviewed, and only trials with sufficiently continuous and unobstructed landmark trajectories throughout the analyzed interval were retained for subsequent processing.

Landmark coordinate trajectories were processed in Python and smoothed using a nine-point, second-order Savitzky–Golay filter before angle calculation. Trunk and shank orientations were defined relative to the horizontal image axis, whereas hip and knee joint angles were calculated as three-point angles formed by the shoulder–hip–knee and hip–knee–ankle landmarks, respectively. The analyzed interval was identified manually by frame-by-frame inspection. The start frame was defined as the first frame showing forward rolling of the front wheel, and the end frame as the frame closest to 180° of crank rotation relative to the initial crank position. A time vector derived from the video frame rate was used for temporal calculations.

Continuous trunk, hip, knee, and shank angle trajectories were time-normalized to 0–100% of the analyzed interval and interpolated to 101 equally spaced points for visualization of the continuous kinematic profiles. Summary kinematic variables used in the subsequent analyses were calculated from the original frame-level trajectories before time normalization. The anatomical landmarks used for markerless kinematic analysis are illustrated in **Figure 2**.

**Figure 2:**
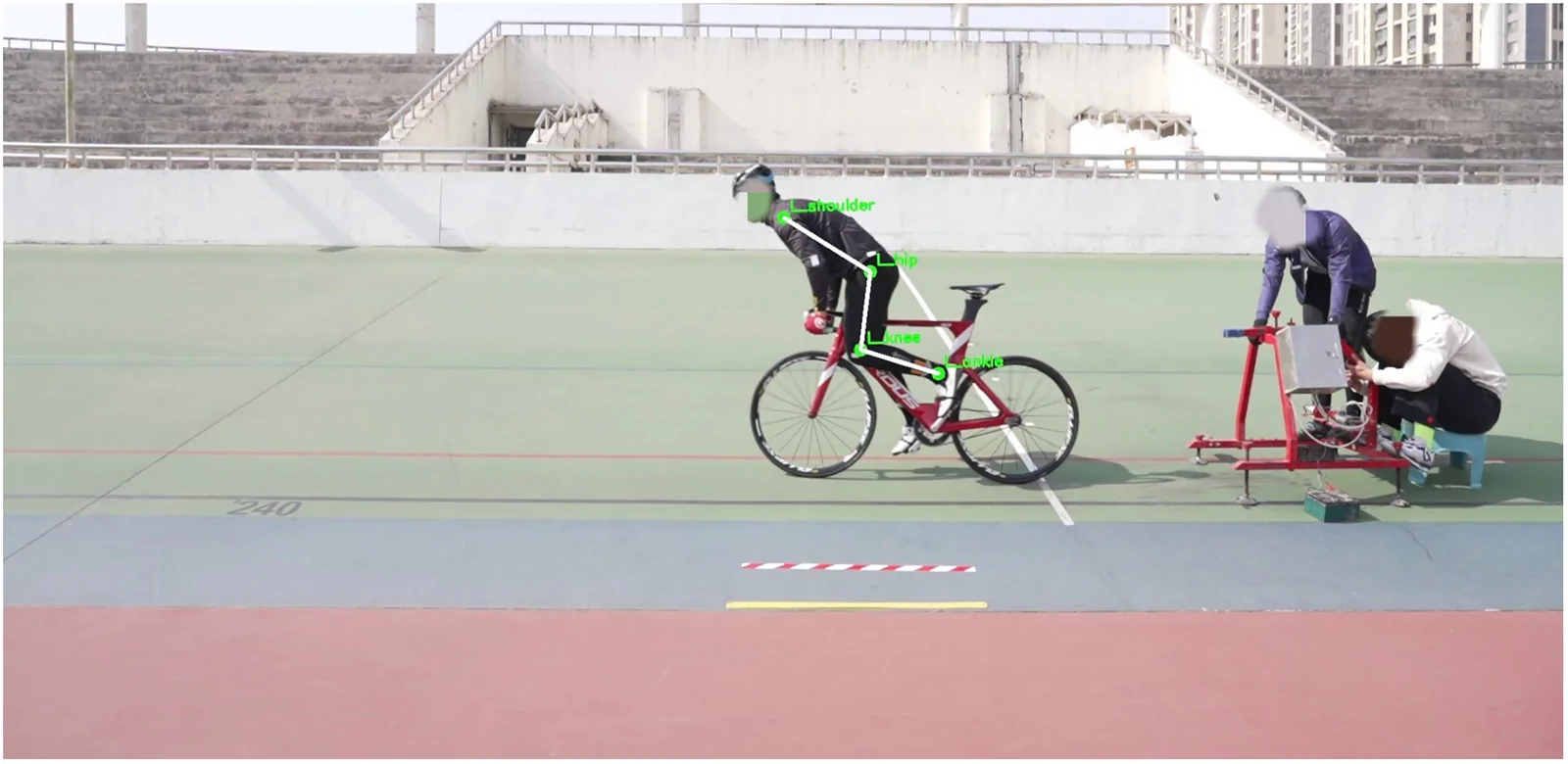
Representative frame illustrating markerless keypoint detection during the standing-start trial.

### 2.6 Kinematic variable extraction

Six summary kinematic variables were extracted from the processed angle trajectories at the original frame-level temporal resolution over the analyzed standing-start interval: first half-crank-cycle duration, trunk angular displacement, hip range of motion (ROM), knee ROM, shank ROM, and peak absolute hip angular velocity. First half-crank-cycle duration was defined as the elapsed time between the start and end frames specified above and was expressed in seconds (s). Hip, knee, and shank ROM were calculated as the difference between the maximum and minimum values of the corresponding angle trajectory within the analyzed interval and were expressed in degrees (°). Trunk angular displacement was defined as the signed net change in trunk angle from the start to the end of the interval (final minus initial angle) and was expressed in degrees (°). Positive values indicated an increase in trunk angle relative to the horizontal image axis, whereas negative values indicated a decrease. Hip angular velocity was estimated by numerical differentiation of the hip-angle trajectory with respect to the frame-derived time vector. Peak absolute hip angular velocity was defined as the maximum absolute angular velocity within the analyzed interval and was expressed in degrees per second (°·s^-^¹).

The kinematic variables were calculated according to the following equations:


$$\text{T}=\frac{{\text{f}}_{\text{end}}-{\text{f}}_{\text{start}}}{{\text{F}}_{\text{s}}}$$

$$\text{ROM}={\text{θ}}_{\text{max}}-{\text{θ}}_{\text{min}}$$

$$△{\text{θ}}_{\text{trunk}}={\text{θ}}_{\text{end}}-{\text{θ}}_{\text{startl}}$$

$${\text{ω}}_{\text{hip},\text{peak}}=\text{max}|\frac{{\text{dθ}}_{\text{hip}}}{\text{dt}}|$$

where T represents first half-crank-cycle duration, ${\text{f}}_{\text{start}}$ and ${\text{f}}_{\text{end}}$ denote the start and end frames, respectively ${\text{F}}_{\text{s}}$ is the video sampling frequency, θ represents the corresponding segment or joint angle, $△{\text{θ}}_{\text{trunk}}$ represents trunk angular displacement, and ${\text{ω}}_{\text{hip},\text{peak}}$ represents peak absolute hip angular velocity.

### 2.7 Trial-to-trial reliability and measurement error

Trial-to-trial reliability of the six kinematic variables was assessed using absolute-agreement intraclass correlation coefficients (ICCs) based on a two-way random-effects model (McGraw and Wong, 1996; Weir, 2005). Single-trial reliability and the reliability of the mean of three trials were expressed as ICC(A,1) and ICC(A,3), respectively, with 95% confidence intervals. Absolute measurement error was quantified using the standard error of measurement (SEM), calculated as the square root of the residual mean square from the repeated-measures ANOVA, and the 95% minimal detectable change (MDC95), calculated as $1.96*\sqrt{2}*SEM$ (Weir, 2005). SEM and MDC95 were also expressed relative to the overall mean of each variable as SEM% and MDC95%, respectively. Within-participant variability was quantified using a log-transformed within-subject coefficient of variation (CV), calculated as $100\sqrt{exp(MS_{error,log})-1}$ (Hopkins, 2000).

Systematic differences among the three trials were assessed using one-way repeated-measures ANOVA. Variables showing a significant overall trial effect were further examined using Holm-adjusted paired t-tests. Wilcoxon signed-rank tests were applied to the corresponding pairwise trial comparisons as a non-parametric sensitivity analysis. For the subsequent association analyses, the three trial values were averaged within each participant to obtain a single participant-level value for each kinematic variable.

### 2.8 Statistical analysis

Statistical analyses were performed using Python (version 3.10.20; Python Software Foundation, Wilmington, DE, USA). Continuous variables are presented as mean ± SD. Data distributions were assessed using the Shapiro–Wilk test, and potential extreme observations were screened using the 1.5 × IQR criterion and standardized scores (|z| ≥ 3). Flagged observations were retained in the primary analyses. Associations between the four physical fitness measures and six participant-level kinematic variables were examined using Spearman’s rank correlation coefficients (ρ), yielding 24 comparisons. Percentile bootstrap 95% CIs were estimated from 10,000 resamples, and the Benjamini–Hochberg false discovery rate (BH-FDR) procedure was applied across all 24 correlations. Associations with raw p < 0.05 were further examined using IQR-based exclusion, where applicable, and leave-one-out sensitivity analyses. All tests were two-sided. Raw p < 0.05 indicated nominal significance, whereas BH-FDR-adjusted p < 0.05 indicated statistical significance after multiple-comparison correction.

## 3 Results

### 3.1 Participant characteristics and physical fitness

Participant characteristics and physical fitness measures are summarized in **Table 1**. The 11 participants had a mean age of 16.09 ± 0.94 years, height of 175.45 ± 6.33 cm, and body mass of 70.73 ± 8.34 kg. Mean standing long jump performance was 249.82 ± 24.41 cm, and relative squat strength was 1.99 ± 0.22 kg·kg^-^¹. Relative mean and peak power were 10.32 ± 0.92 and 16.76 ± 1.60 W·kg^-^¹, respectively.

**Table 1:** Participant characteristics and physical fitness measures (n = 11).

Variable	Mean ± SD	Range
Age (years)	16.09 ± 0.94	15–18
Height (cm)	175.45 ± 6.33	168–184
Body mass (kg)	70.73 ± 8.34	57–87
Standing long jump (cm)	249.82 ± 24.41	203–283
Relative squat strength (kg·kg^-^¹)	1.99 ± 0.22	1.75–2.36
Relative mean power (W·kg^-^¹)	10.32 ± 0.92	8.46–11.67
Relative peak power (W·kg^-^¹)	16.76 ± 1.60	14.45–19.06

### 3.2 Trial-to-trial reliability of standing-start kinematic variables

Trial-to-trial reliability estimates for the six standing-start kinematic variables are presented in **Table 2**. ICC(A,1) values ranged from 0.587 to 0.901, whereas ICC(A,3) values ranged from 0.810 to 0.965. Measurement-error indices, including SEM, MDC95, and CV, are also reported in **Table 2**.

**Table 2:** Trial-to-trial reliability and measurement error of standing-start kinematic variables across three repeated trials (n = 11).

Variable	Trial 1 (mean ± SD)	Trial 2 (mean ± SD)	Trial 3 (mean ± SD)	ICC(A,1) (95% CI)	ICC(A,3) (95% CI)	SEM	SEM (%)	MDC95	MDC95 (%)	CV (%)	Trial effect p
First half-crank-cycle duration (s)	1.349 ± 0.152	1.324 ± 0.149	1.308 ± 0.153	0.894 (0.74–0.97)	0.962 (0.90–0.99)	0.047	3.56	0.131	9.86	3.56	0.148
Trunk angular displacement (°)	8.04 ± 5.45	6.49 ± 5.51	6.86 ± 6.28	0.744 (0.46–0.91)	0.897 (0.72–0.97)	2.93	41.06	8.12	113.83	83.65	0.447
Hip ROM (°)	49.04 ± 6.95	46.33 ± 7.52	48.95 ± 7.18	0.746 (0.47–0.91)	0.898 (0.72–0.97)	3.51	7.30	9.73	20.23	7.42	0.147
Knee ROM (°)	61.84 ± 9.54	54.65 ± 7.23	60.42 ± 8.70	0.587 (0.21–0.85)	0.810 (0.44–0.95)	4.78	8.10	13.24	22.45	8.49	0.005
Shank ROM (°)	52.29 ± 8.18	50.00 ± 6.31	52.14 ± 7.15	0.746 (0.47–0.91)	0.898 (0.73–0.97)	3.61	7.01	10.00	19.42	7.66	0.273
Peak absolute hip angular velocity (°·s^-^¹)	284.9 ± 75.9	285.1 ± 84.6	283.8 ± 88.7	0.901 (0.75–0.97)	0.965 (0.90–0.99)	27.3	9.60	75.7	26.61	9.90	0.993

Values for individual trials are presented as mean ± SD. ICC(A,1), single-measure absolute-agreement intraclass correlation coefficient; ICC(A,3), absolute-agreement intraclass correlation coefficient for the mean of three trials; CI, confidence interval; SEM, standard error of measurement; MDC95, minimal detectable change at the 95% confidence level; CV, log-transformed within-subject coefficient of variation; ROM, range of motion. Trial effects were assessed using one-way repeated-measures ANOVA. Significant overall trial effects were followed by Holm-adjusted paired t-tests; Wilcoxon signed-rank tests were used as a non-parametric sensitivity analysis.

A significant overall trial effect was observed for knee ROM, F(2,20) = 6.999, p = 0.005. Holm-adjusted paired comparisons showed differences between T1 and T2 (adjusted p = 0.040) and between T2 and T3 (adjusted p = 0.043), whereas T1 and T3 did not differ (adjusted p = 0.362). The Wilcoxon signed-rank sensitivity analysis showed the same pattern of significance. No significant trial effects were observed for first half-crank-cycle duration, trunk angular displacement, hip ROM, shank ROM, or peak absolute hip angular velocity (all p > 0.05).

### 3.3 Continuous kinematic profiles

**Figure 3** presents the continuous kinematic trajectories across the normalized standing-start interval. The trunk forward angle increased from 24.54 ± 4.52° at 0% to a maximum of 41.21 ± 4.39° at 58%, and subsequently decreased to 30.72 ± 5.20° at 100%. The hip angle increased from 99.54 ± 5.89° to a maximum of 109.70 ± 8.26° at 42%, decreased to a minimum of 67.75 ± 5.50° at 77%, and ended at 88.99 ± 5.92°. The knee angle decreased from 125.12 ± 8.60° to a minimum of 71.56 ± 4.06° at 65%, before increasing to 118.71 ± 7.31° at 100%. The shank angle increased from −50.12 ± 5.11° to a maximum of −13.98 ± 5.05° at 56%, and subsequently decreased to −60.45 ± 6.13° at 100%.

**Figure 3:**
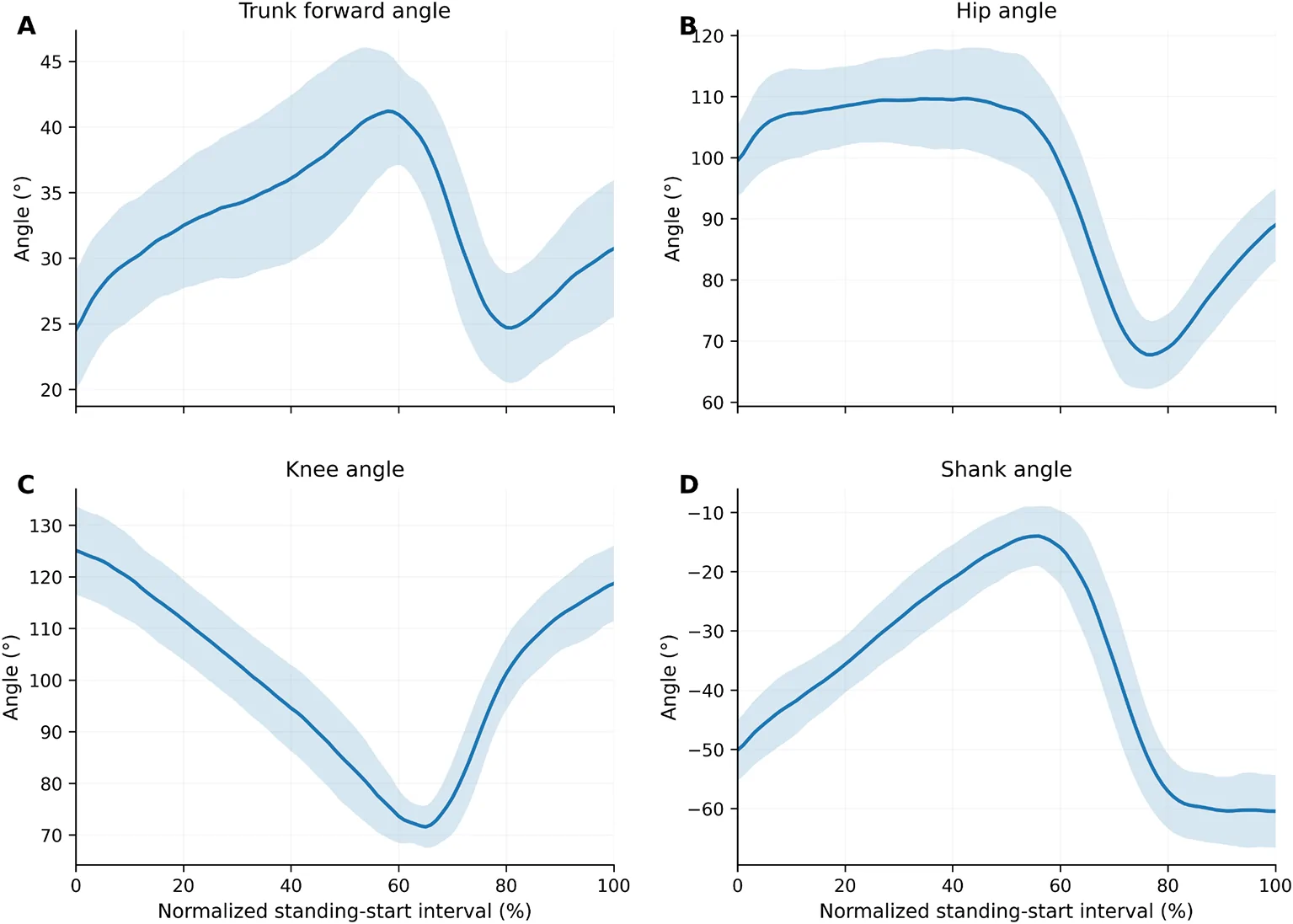
Continuous trunk and lower-limb kinematic trajectories during the standing-start interval. Group-mean trajectories for **(A)** trunk forward angle, **(B)** hip angle, **(C)** knee angle, and **(D)** shank angle across the normalized standing-start interval (0–100%). Solid lines represent the group mean and shaded areas represent ±1 SD across participants (n = 11). Each participant-level trajectory was obtained by averaging the three valid standing-start trials before calculation of the group-level mean and standard deviation.

### 3.4 Associations between physical fitness and standing-start kinematics

Spearman’s rank correlation analysis was used to examine associations between physical fitness and standing-start kinematic variables (**Figure 4**). Relative mean power was positively correlated with knee ROM (ρ = 0.700, raw p = 0.0165), representing the only association that reached nominal statistical significance. Among the remaining associations, relative peak power was positively correlated with knee ROM (ρ = 0.527), while relative squat strength was positively correlated with peak absolute hip angular velocity (ρ = 0.427). The absolute correlation coefficients for the remaining associations did not exceed 0.27. None of the 24 associations remained statistically significant after Benjamini–Hochberg false discovery rate correction. Full results for all 24 prespecified correlations, including Spearman’s ρ, bootstrap 95% confidence intervals, raw p values, and BH-FDR-adjusted p values, are provided in **Supplementary Table S1**.

**Figure 4:**
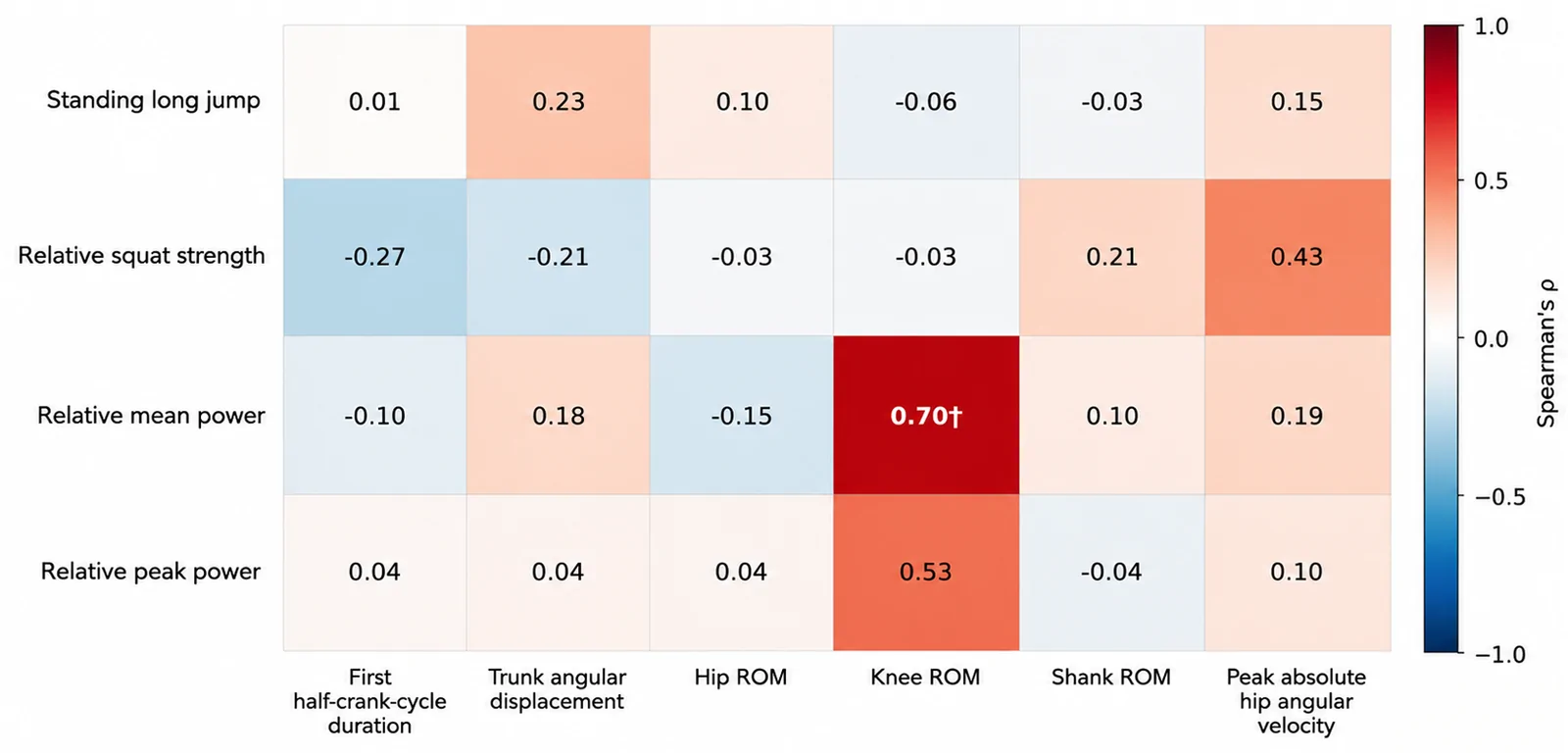
Spearman’s rank correlations between physical fitness and standing-start kinematic variables (n = 11). Values represent Spearman’s rank correlation coefficients (ρ). Positive and negative correlations are represented by red and blue, respectively, with color intensity indicating the magnitude of the correlation coefficient. † indicates raw p < 0.05. No association remained statistically significant after Benjamini–Hochberg false discovery rate correction.

## 4 Discussion

### 4.1 Continuous kinematic characteristics

Using a cross-sectional design, the present study examined continuous sagittal-plane kinematics during the early standing-start phase in adolescent male track cyclists, focusing on the temporal organization of trunk and lower-limb motion. Distinct temporal patterns were observed across the trunk, hip, knee, and shank, reflecting differences in segmental motion throughout the standing-start interval. Previous standing-start studies have reported contributions from the trunk and lower limbs, together with increased trunk and upper-limb muscle activation during the first pedal stroke in the standing position (Zhang et al., 2015; Padulo et al., 2016). Cycling-specific research has also shown temporal relationships among joint and segment motions, indicating that cycling movement is not adequately characterized by isolated joint actions (Sides and Wilson, 2012). The present kinematic patterns are therefore consistent with the multi-segment nature of standing-start execution described in previous cycling research.

The temporal differences among segments also highlight information that is not fully represented by discrete kinematic measures. In the present study, continuous trajectories captured changes in trunk and lower-limb motion across the normalized standing-start interval, whereas ROM and peak angular velocity summarized specific features of the movement. Whole-curve analyses in cycling have similarly shown that restricting kinematic assessment to selected time points may overlook differences occurring within specific phases of the movement cycle (Galindo-Martínez et al., 2021). Coordination studies further indicate that temporal relationships between joints can distinguish movement patterns even when differences in individual joint angles are limited (Chapman et al., 2009; Sides and Wilson, 2012). Continuous and discrete measures therefore provide different but complementary information for describing standing-start kinematics.

The temporal patterns observed during the standing start are also likely to depend on the mechanical and technical constraints of the cycling task. Cadence and inertial load can modify the timing and magnitude of muscle activation, while changes in pedaling technique alter neuromuscular recruitment and cycling efficiency (Baum and Li, 2003; Cannon et al., 2007). Joint motion is similarly influenced by cycling conditions and bicycle configuration. During maximal cycling, changes in pedaling rate alter joint-specific mechanics and hip and ankle excursion, whereas knee excursion may remain relatively stable (McDaniel et al., 2014). Saddle height and crank length can also modify hip and knee kinematics, including joint flexion and ROM (Bini et al., 2012; Ferrer-Roca et al., 2017). These factors indicate that the continuous profiles identified in the present study represent movement patterns under the specific testing conditions rather than a fixed technical model of the standing start. Their relationship with mechanical effectiveness and start performance requires further examination using concurrent kinetic and performance measures.

### 4.2 Trial-to-trial reliability of standing-start kinematics

The interpretation of standing-start kinematics also depends on whether the derived measures remain consistent across repeated trials. Standardized acquisition and analysis are central to the reproducibility of cycling kinematic assessment, particularly when joint and segment measures are used to characterize individual movement patterns (Priego-Quesada et al., 2024). In the present study, three-trial mean reliability was higher than single-trial reliability, with ICC(A,3) ranging from 0.810 to 0.965 across the six kinematic variables. Averaging repeated trials provided a more stable estimate of each athlete’s standing-start kinematics and reduced the influence of variation associated with a single trial. This supports the use of three-trial mean values for the subsequent participant-level analysis rather than relying on an individual standing-start attempt.

Despite the generally consistent reliability across repeated trials, knee ROM differed from the other kinematic variables by showing a significant trial effect, indicating systematic within-session variation across repeated trials. Such variation is plausible because lower-limb mechanics in cycling are sensitive to task demands and movement organization. McDaniel et al. (2014) showed that changes in pedaling rate produced joint-specific adaptations in lower-limb mechanics and excursion during maximal cycling, while Sides and Wilson (2012) reported that coordination variability and inter-joint phase relationships differed with cadence and cycling experience. Similar evidence indicates that neuromuscular coordination changes with cadence and inertial load (Baum and Li, 2003), further illustrating that repeated cycling movements are not mechanically invariant. The trial effect observed for knee ROM may therefore reflect within-session variation in movement execution, measurement-related variability, or a combination of both. This variability warrants caution when knee ROM is interpreted as an individual characteristic of standing-start technique.

### 4.3 Associations between physical fitness and standing-start kinematics

With the standing-start kinematic measures represented by three-trial mean values, the association analysis examined whether individual differences in physical fitness were reflected in movement characteristics during the early start phase. No association remained statistically significant after correction for multiple comparisons, indicating limited evidence for consistent relationships between the measured physical capacities and individual kinematic variables. This finding does not diminish the established role of strength and power in sprint cycling. Maximal and relative power are important determinants of sprint-cycling performance and early acceleration, while maximal strength and rapid force-development capacity contribute to sprint-cycling force and torque production (Douglas et al., 2021; Connolly et al., 2023). Recent evidence has also shown strong associations between physical capacities and standing-start acceleration performance, a broader performance outcome than the individual kinematic characteristics examined here (Michalik et al., 2026). Evidence from track sprint cyclists further indicates that strength gains can be accompanied by changes in cycling power and intermuscular coordination, although their transfer to cycling mechanics is task specific (Burnie et al., 2022). The present results therefore suggest that physical capacity and standing-start kinematics should not be expected to correspond on a one-to-one basis.

Within this limited association pattern, a nominal positive correlation was observed between relative mean power and knee ROM, although the relationship did not remain significant after FDR correction. A broader biomechanical context for interpreting this pattern is provided by evidence that lower-limb joint motion and mechanical contributions vary with power demand, cadence, and movement conditions (McDaniel et al., 2014; Bini et al., 2016). Changes in cycling intensity have also been associated with adaptations in lower-limb kinematics, indicating that joint motion is responsive to the mechanical demands of the task (Pouliquen et al., 2021). Knee ROM, however, is likely influenced by multiple factors beyond physical capacity. Bicycle configuration can alter hip and knee kinematics, with changes in saddle height and crank length affecting joint position and excursion (Bini et al., 2012; Ferrer-Roca et al., 2017). The nominal association should therefore be interpreted cautiously, as knee motion may reflect multiple physical and task-related influences rather than power capacity alone. Given the loss of statistical significance after FDR correction, this association should be considered exploratory and requires confirmation in a larger sample.

The overall limited association pattern may reflect the multifactorial nature of cycling movement. Physical capacity represents only one component of movement execution, whereas joint and segmental kinematics are also shaped by task demands, pedaling strategy, and inter-joint coordination. Cadence and mechanical demand can redistribute joint-specific power and alter lower-limb mechanics (Aasvold et al., 2019; McDaniel et al., 2014), while changes in pedaling technique and cycling experience are associated with differences in neuromuscular and coordinative organization (Cannon et al., 2007; Chapman et al., 2009; Sides and Wilson, 2012). Kinematic patterns may also vary with the athlete’s physiological state, as fatigue-related changes in muscle activity have been accompanied by changes in trunk and lower-limb kinematics (Dingwell et al., 2008). The temporal specificity of the fitness assessment should also be considered, as the 30-s cycling test used here reflects a longer-duration effort than the early standing-start phase examined, whereas shorter-duration cycling power has been associated with standing-start acceleration performance (Michalik et al., 2026). Taken together, these movement- and assessment-related considerations provide a plausible context for the weak and variable-specific associations observed in the present study, in which differences in physical fitness were not consistently reflected in individual kinematic measures. Standing-start technique may therefore be better characterized by considering physical capacity and movement organization as related but distinct components of performance.

### 4.4 Practical implications

From a practical perspective, the present findings support the combined assessment of physical capacity and standing-start technique in adolescent track cyclists. Strength and power remain important components of sprint-cycling performance, particularly during the high-force demands of early acceleration (Douglas et al., 2021). Evidence from track sprint cyclists further indicates that improvements in strength can be accompanied by changes in cycling power and neuromuscular coordination, although the transfer of strength gains to cycling mechanics is task specific (Burnie et al., 2022). Accordingly, physical fitness measures and standing-start kinematics should be considered complementary rather than interchangeable sources of information. Monitoring changes in strength and power alongside individual kinematic characteristics may provide complementary information for evaluating an athlete’s development without assuming that changes in physical fitness are accompanied by corresponding technical adaptations.

Kinematic monitoring may be particularly useful when repeated assessments are used to track how an individual athlete organizes the standing start over time. The first pedal stroke involves contributions from the lower limbs, trunk, and upper body, indicating that technical assessment should extend beyond isolated joint measures (Padulo et al., 2016). Changes in physical capacity may not necessarily be reflected in uniform changes in individual joint kinematics, and cycling-specific coordination may provide additional information on technical adaptation (Burnie et al., 2022). Continuous trajectories and selected discrete measures may therefore provide a useful basis for monitoring within-athlete changes in movement timing and segmental organization across training periods. Given the absence of robust fitness–kinematic associations in the present study, these measures are better suited to individualized technical monitoring than to defining universal target values for ROM, joint angles, or movement timing.

### 4.5 Limitations

Several limitations should be considered when interpreting these findings. The small sample of 11 adolescent male track cyclists limits the precision and broader generalizability of the results, and the cross-sectional, exploratory design precludes causal inference; therefore, the observed associations require confirmation in larger samples. Although approximately 3 h separated the physical-fitness and standing-start sessions, residual effects of the earlier testing cannot be completely excluded. In addition, the fixed Wattbike resistance may have imposed different relative mechanical demands across participants, and the 30-s sprint test may not fully reflect the short-duration demands of the analyzed standing-start phase. Individually selected initial crank positions may also have contributed to between-participant variability. The two-dimensional sagittal-plane analysis could not characterize out-of-plane motion or three-dimensional coordination, and the RTMPose-based pipeline was not concurrently validated against a criterion reference system (Peebles et al., 2021; Priego-Quesada et al., 2024). Finally, the absence of direct kinetic or standing-start performance measures limits interpretation of whether the observed kinematic characteristics were associated with actual start performance.

## 5 Conclusions

Standing-start kinematic measures showed generally high reliability when averaged across three trials. No fitness–kinematic association remained statistically significant after BH-FDR correction, although the strongest correlations were observed for relative mean power with knee ROM, relative peak power with knee ROM, and relative squat strength with peak absolute hip angular velocity. These associations remain exploratory and require confirmation in larger samples.

## Data Availability

The raw data supporting the conclusions of this article will be made available by the authors, without undue reservation.
