# Supplementary material for: Relationships between physical fitness and early standing-start kinematic characteristics in adolescent male track cyclists
Source: Front Physiol. 2026 Oct 8;17:1948251. doi: 10.3389/fphys.2026.1948251 (PMC13686819; doi:10.3389/fphys.2026.1948251)
Supplement: Supplementary file 1 [file Table1.docx]

**Supplementary Table S1.** Spearman correlations between physical fitness and standing-start kinematic variables

| **Physical-fitness variable** | **Kinematic variable** | **Spearman ρ** | **Raw p** | **BH-FDR-adjusted p** | **Bootstrap 95% CI** |
| --- | --- | --- | --- | --- | --- |
| Standing long jump (cm) | First half-crank-cycle duration (s) | 0.014 | 0.9681 | 0.9681 | [-0.640, 0.687] |
| Standing long jump (cm) | Trunk angular displacement (°) | 0.228 | 0.5005 | 0.9681 | [-0.516, 0.882] |
| Standing long jump (cm) | Hip ROM (°) | 0.096 | 0.7796 | 0.9681 | [-0.687, 0.746] |
| Standing long jump (cm) | Knee ROM (°) | -0.059 | 0.8627 | 0.9681 | [-0.704, 0.664] |
| Standing long jump (cm) | Shank ROM (°) | -0.032 | 0.9258 | 0.9681 | [-0.613, 0.626] |
| Standing long jump (cm) | Peak absolute hip angular velocity (°·s⁻¹) | 0.150 | 0.6590 | 0.9681 | [-0.549, 0.738] |
| Relative squat strength (kg·kg⁻¹) | First half-crank-cycle duration (s) | -0.269 | 0.4242 | 0.9681 | [-0.862, 0.522] |
| Relative squat strength (kg·kg⁻¹) | Trunk angular displacement (°) | -0.209 | 0.5372 | 0.9681 | [-0.907, 0.579] |
| Relative squat strength (kg·kg⁻¹) | Hip ROM (°) | -0.027 | 0.9366 | 0.9681 | [-0.827, 0.682] |
| Relative squat strength (kg·kg⁻¹) | Knee ROM (°) | -0.027 | 0.9366 | 0.9681 | [-0.641, 0.654] |
| Relative squat strength (kg·kg⁻¹) | Shank ROM (°) | 0.209 | 0.5372 | 0.9681 | [-0.615, 0.846] |
| Relative squat strength (kg·kg⁻¹) | Peak absolute hip angular velocity (°·s⁻¹) | 0.427 | 0.1899 | 0.9681 | [-0.378, 0.943] |
| Relative mean power (W·kg⁻¹) | First half-crank-cycle duration (s) | -0.105 | 0.7591 | 0.9681 | [-0.771, 0.517] |
| Relative mean power (W·kg⁻¹) | Trunk angular displacement (°) | 0.182 | 0.5926 | 0.9681 | [-0.532, 0.915] |
| Relative mean power (W·kg⁻¹) | Hip ROM (°) | -0.155 | 0.6500 | 0.9681 | [-0.692, 0.537] |
| Relative mean power (W·kg⁻¹) | Knee ROM (°) | 0.700 | 0.0165 | 0.3953 | [0.127, 0.972] |
| Relative mean power (W·kg⁻¹) | Shank ROM (°) | 0.100 | 0.7699 | 0.9681 | [-0.775, 0.748] |
| Relative mean power (W·kg⁻¹) | Peak absolute hip angular velocity (°·s⁻¹) | 0.191 | 0.5739 | 0.9681 | [-0.505, 0.731] |
| Relative peak power (W·kg⁻¹) | First half-crank-cycle duration (s) | 0.041 | 0.9047 | 0.9681 | [-0.636, 0.705] |
| Relative peak power (W·kg⁻¹) | Trunk angular displacement (°) | 0.045 | 0.8944 | 0.9681 | [-0.656, 0.718] |
| Relative peak power (W·kg⁻¹) | Hip ROM (°) | 0.036 | 0.9155 | 0.9681 | [-0.547, 0.657] |
| Relative peak power (W·kg⁻¹) | Knee ROM (°) | 0.527 | 0.0956 | 0.9681 | [-0.194, 0.943] |
| Relative peak power (W·kg⁻¹) | Shank ROM (°) | -0.036 | 0.9155 | 0.9681 | [-0.777, 0.738] |
| Relative peak power (W·kg⁻¹) | Peak absolute hip angular velocity (°·s⁻¹) | 0.100 | 0.7699 | 0.9681 | [-0.620, 0.649] |

Note: n = 11 for all correlations. ρ, Spearman’s rank correlation coefficient; CI, confidence interval; ROM, range of motion; BH-FDR, Benjamini–Hochberg false discovery rate. Bootstrap 95% CIs were based on 10,000 resamples. Raw p values were adjusted across the 24 prespecified correlation tests using the BH-FDR procedure. No association remained statistically significant after correction for multiple comparisons.
